# A multi-stage 130 m/s reluctance linear electromagnetic launcher

**DOI:** 10.1038/s41598-022-27022-z

**Published:** 2023-01-05

**Authors:** Moshe Einat, Yafit Orbach

**Affiliations:** Department of Electrical and Electronic Engineering, Faculty of Engineering, Ariel University, Ariel, Israel

**Keywords:** Electrical and electronic engineering, Aerospace engineering

## Abstract

Moon launching capabilities are vital for space program development. Especially important is the capability to launch without fuels or disposable elements since bringing supplies to the moon is complicated and expensive. An electric system would have the benefit of using solar or nuclear-based unlimited electrical energy. In this paper, such an electrical launching system is suggested—a reluctance coilgun launcher with multi acceleration stages. It has the benefit of simplicity and longer lifetime compared to other electrical launchers. In this paper, a successful implementation of a multi-stage reluctance launcher is presented that reaches the highest reported launching speeds from a reluctance coilgun. Moreover, a method to successfully add more and more stages is presented. Based on this method, an electrical launcher to be used for launching from the moon can be designed.

In the year 1865, the science fiction novel "" by Jules Verne described the concept of a gun shot 'From the Earth to the Moon'. This is still not realistic today. However, perhaps a gun shot from the moon can become a reality?

Future human exploration of space in general and the moon^1–3^ in particular will require the development of in-situ energized launching capabilities. There is research aiming to extract oxygen and metals from the moon's soil^4,5^ that can be used for refueling rockets in space, but a solar based electrical launching system may be the most practical solution to launch objects (including the extracted oxygen) from the moon to space. This understanding led to the design and analysis of electromagnetic launchers (EMLs)^6–9^ that will launch from the moon or space relying only on solar energy, without any fuels or oxygen. Space missions to Mars^10^ and outer space are also considered in two stages where the second stage is from space. Nanosatellites (cubesat) are important parts of modern space research and they are launched from the space vehicle to orbit. Therefore, there is great interest in developing an electrical launcher configuration that will meet the space requirements.

The main EMLs are the rail gun and the coil gun^11^, with the coilgun divided to induction and reluctance configurations. Rail guns were suggested for lunar and space missions^12–15^, but a critical difficulty is the damage to the rails during the launch, with new rails needed frequently. The induction coil gun was also suggested^7–9^ and it has the benefit of no damage during the launch and endless lifetime. The reluctance coil gun that is based on the magnetic force^16^ is even simpler to use, but could not be considered as a candidate for space due to the low launching speed. Even though there is no conceptual launch speed limit in the reluctance launcher, experimental studies demonstrated only low launching speeds. Coilguns (of both types) have the advantage of the possibility to cascade accelerating stages to increase the speed more and more. Even so, the reluctance coil gun experimentally demonstrated relatively low launching speeds. Experiments showed that the launching mass and energy can be extended, but the speed remained low. Research^17–35^ has shown results of simulations and related experiments aimed at increasing launching velocity and energy (Fig. 1). A split coil design was presented by Manzoor et al.^19^, demonstrating 36 m/s and 6 J launching energy. Zhu et al.^20^ demonstrated a hybrid coilgun with conductive rails and sliding contact brushes, similar to railgun. 19.8 m/s launching velocity of 0.3 kg projectile were obtained, enduring the sliding contact disadvantage. Kim et al.^18^ presented an accurate simulation and measured 36.6 m/s with 0.39 J. Makowski et al.^21^ demonstrated a hybrid gun with air gun section, coilgun section and finally railgun. The velocity after the coilgun section was 26 m/s and the final velocity was 30.8 m/s. Citak et al.^24^ presented voltage and solenoid optimizations resulting at 16.5 m/s. Rivas-Camacho et al.^25^ presented coilgun with an inductive power source reaching 8.96 m/s. Deng et al.^29,34^ showed 30 J record launching energy of coilgun by extending the projectile mass, shortening the electrical pulse, and optimizing the projectile material and shape. Akay et al.^30^ demonstrated a 17.1 m/s.

**Figure 1:**
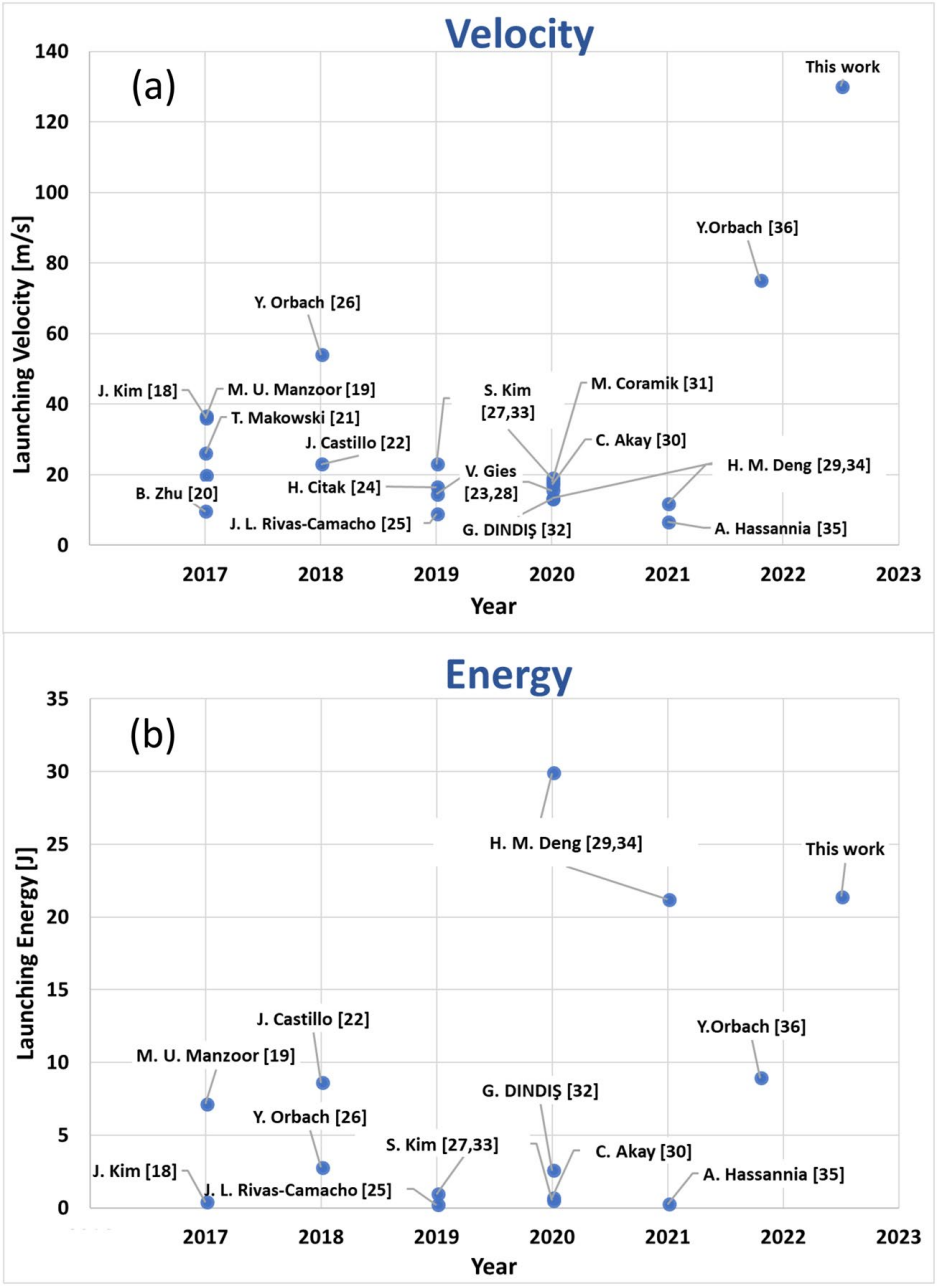
A collection of the launching velocity (**a**) and launching energy (**b**) in the last 5 years of experiments. Every dot represents launching velocities/ energy of an experiment that is in the attached reference name.

4-stage coilgun without capacitor. Another 4-stage coilgun was reported by Coramik et al.^31^, aiming to increase barrel exit velocity, obtaining 18 m/s. Kim et al.^27^ reported a coilgun where the shape of the projectile was optimally designed to obtain high magnetic force and a small drag coefficient, 23 m/s was measured. Yet, it can be clearly seen that typical velocity values are below ~ 35 m/s. In 2018, a 54 m/s with the use of a single stage^26^ and, in 2022, a 75 m/s with 2 stages were reported^36^.

Typical mass of a projectile among the various systems is few grams (2–11 g) with diameter of few millimeters. However, Extending the energy of the projectile was demonstrated by increasing the projectile mass to much larger values. Deng et al.^29,34^ demonstrated 340 g projectile with 30 mm diameter and 60 mm length. Gies et al.^23,28^ used 690 g plunger with 25 mm diameter and 115 mm length, that was used to push load without launching. Nevertheless, for launching applications high energy is not enough, the velocity must be extended as well.

In this study the projectile is kept small (2.5 g) but the velocity is greatly extended. A multi stage reluctance coil gun is explored both theoretically and experimentally. A simulation was used for optimized control timing of the stages and a redesign of the electrical circuit was done to shorten the current pulses. A successful model is presented where the velocity is increased from stage to stage and a record velocity of 130 m/s is measured. The method is modular and extension of the gun by adding more and more stages is practical. This study positions the reluctance coil gun as a realistic EML candidate for future lunar and space missions. Once the practical way to unlimitedly cascade stages to the reluctance multistage coil gun is found, lunar implementation with this technique can be considered.

## The reluctance coil gun

The reluctance coil gun includes a winding coil and a ferromagnetic cylinder projectile. The projectile is attracted to the center of the coil, where the reluctance (resistance against the magnetic field) is minimum. The force propelling the projectile can be expressed by the force acting on the ferromagnetic material in the magnetic field^16^.

$$\vec{F} = \nabla \left( {\vec{m} \cdot {\vec{\text{B}}}} \right)$$

The force $\overrightarrow{F}$ depends on the gradient of magnetic field $\overrightarrow{\mathrm{B}}$ and magnetic moment $\overrightarrow{m}$. The projectile is accelerated in the direction of the magnetic field gradient; therefore, if the projectile will pass the coil center while the current is still running in the coil, the projectile will be pulled back into the coil. Thus, right timing and current pulse duration have a great impact on the projectile velocity.

Launching velocity is important characteristic of the launcher. Increasing the reluctance coilgun launching velocity is the goal of many works. However, as seen above, in spite of widespread efforts, increasing the velocity has been limited. Other parameters such as energy and efficiency were improved, but the velocity is still challenging. One reason is that as the velocity grows the projectile passes through the accelerating stage in a shorter time. Therefore, the current pulse must be shortened. But this goes against the nature of solenoids. Having a strong magnetic field requires higher inductance that leads to longer pulses. So, the electronic circuit must support a strong and short current pulse running through the solenoids. Another reason is the difficulty to establish an accurate simulation that predicts accurately at high speeds. For low speeds, many simulations work well, but for high speeds the inter-dependence of the parameters and the nonlinearity of the effects lead to faulty results. After obtaining a record result for a single stage^26^, and demonstrating successful cascading of two stages^36^, in this paper a multi stage reluctance launcher is presented. The electrical circuit design supports short pulses and energy recovery, and the simulation takes into account all the complexity of the system. This design method supports the upscaling of the coilgun and, therefore, makes it a candidate for space and lunar EML.

## Electrical circuit

**Figure 2:**
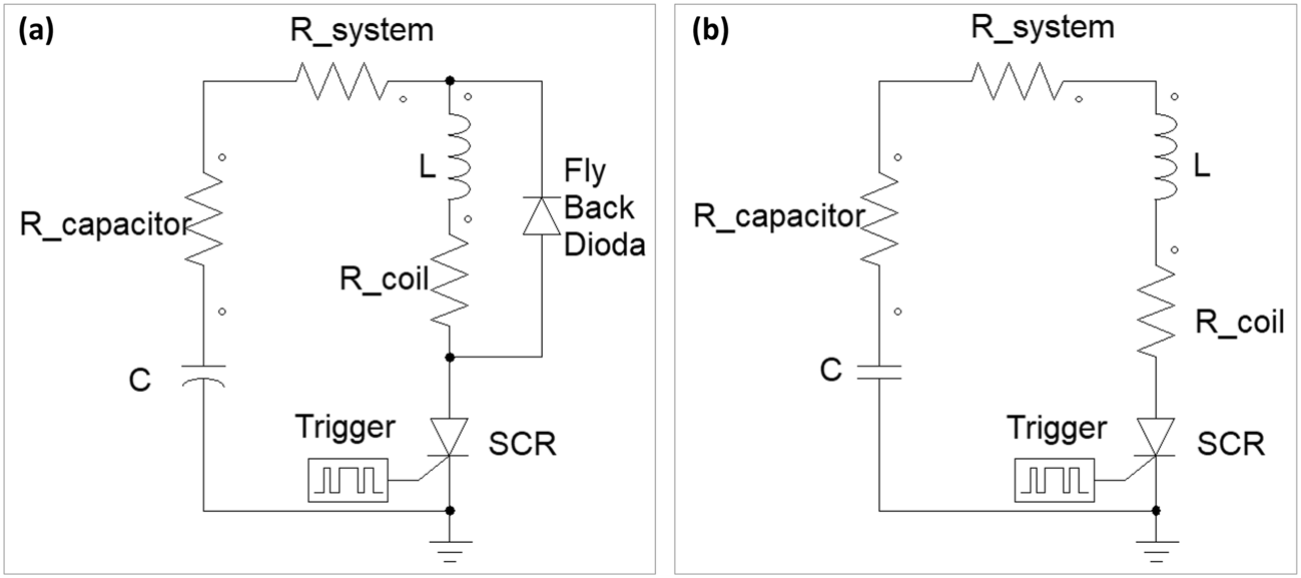
Electrical schematic diagram of (**a**) typical ignition circuit and (**b**) fast discharge energy recycling ignition circuit.

The launcher performance depends on the driving ignition circuit, pulse duration, current amplitude and electrical efficiency. For higher efficiency, an RLC (resistor, inductor, capacitor) underdamped response is required. Low resistance in the circuit will lead to a larger energy transfer from the capacitor to the coil, allowing higher current and therefore a stronger magnetic field. The typical circuit used for coilguns is seen schematically in Fig. 2a. The circuit includes a capacitor with equivalent series resistance (ESR), a Silicon Controlled Rectifier (SCR) as a current switching element, a coil, and fly-back diode for coil discharging. R system is the resistance of the SCR, wires and connectors in the circuit.

When the trigger is given to the SCR the capacitor is discharged and a current is developed in the coil. When maximal current is reached the voltage of the coil is reversed and the flyback diode is therefore turned on. As a result, the coil current is mostly directed to the diode and slowly discharges while dissipating the energy on the diode and coil resistances. The current in the diode increase rapidly and the current in the SCR drops simultaneously, while the coil current slowly decays. Only when the SCR current drops below the minimal holding current the SCR is closed.

The disadvantage of this circuit is the relative long discharge time of the current in the coil that can lead to slowing down of the projectile. Many works have focused on various ways to cut off the last part of the current when the projectile reaches the coil center. In order to shorten the pulse duration, in the presented setup a different discharge circuit was used, with the electrolytic unipolar capacitor replaced by a bipolar capacitor (Fig. 2b) and the fly-back diode removed. Once the capacitor is discharged, the current in the new circuit will not be directed to the fly-back diode. Instead, it will continue to the capacitor recharging it in a reversed polarity, leading to a fast discharge of the coil current in view of the high reverse voltage developed on the capacitor. A shorter pulse will end before the projectile pass the coil center and prevent the projectile pull-back by the residual current. Simultaneously, energy recycling will be obtained.

**Figure 3:**
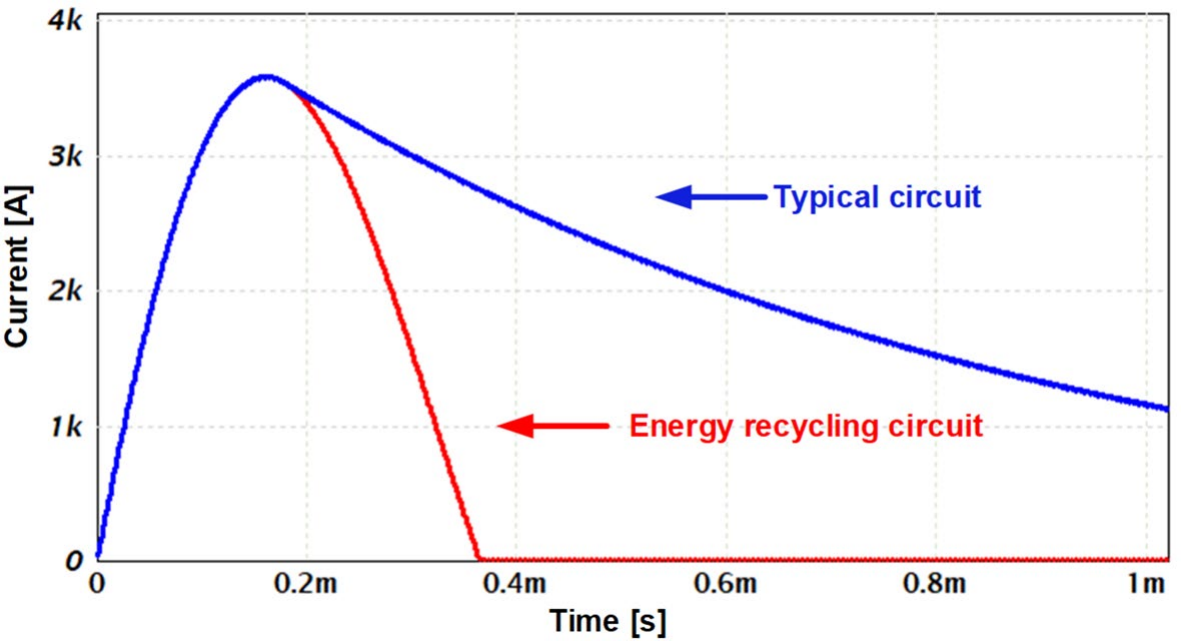
Comparison between the current pulses obtained from the typical circuit (blue curve) and the fast discharge circuit (red curve).

An electrical simulation comparison of the current obtained in the two circuits is seen in Fig. 3. The 'typical' circuit includes a 590 µF film capacitor with Equivalent Series Resistance (ESR) of 2.1 mΩ charged to 900 V, SCR, coil (22 µH, 30 mΩ) and a fly-back diode for coil discharging. The R system is taken as 39 mΩ. The fast discharge circuit is based on the same components without the discharging diode. The coil parameters such as induction and resistance were selected while maintaining an underdamped response. As seen, in the fast discharge circuit a much shorter pulse is obtained.

**Figure 4:**
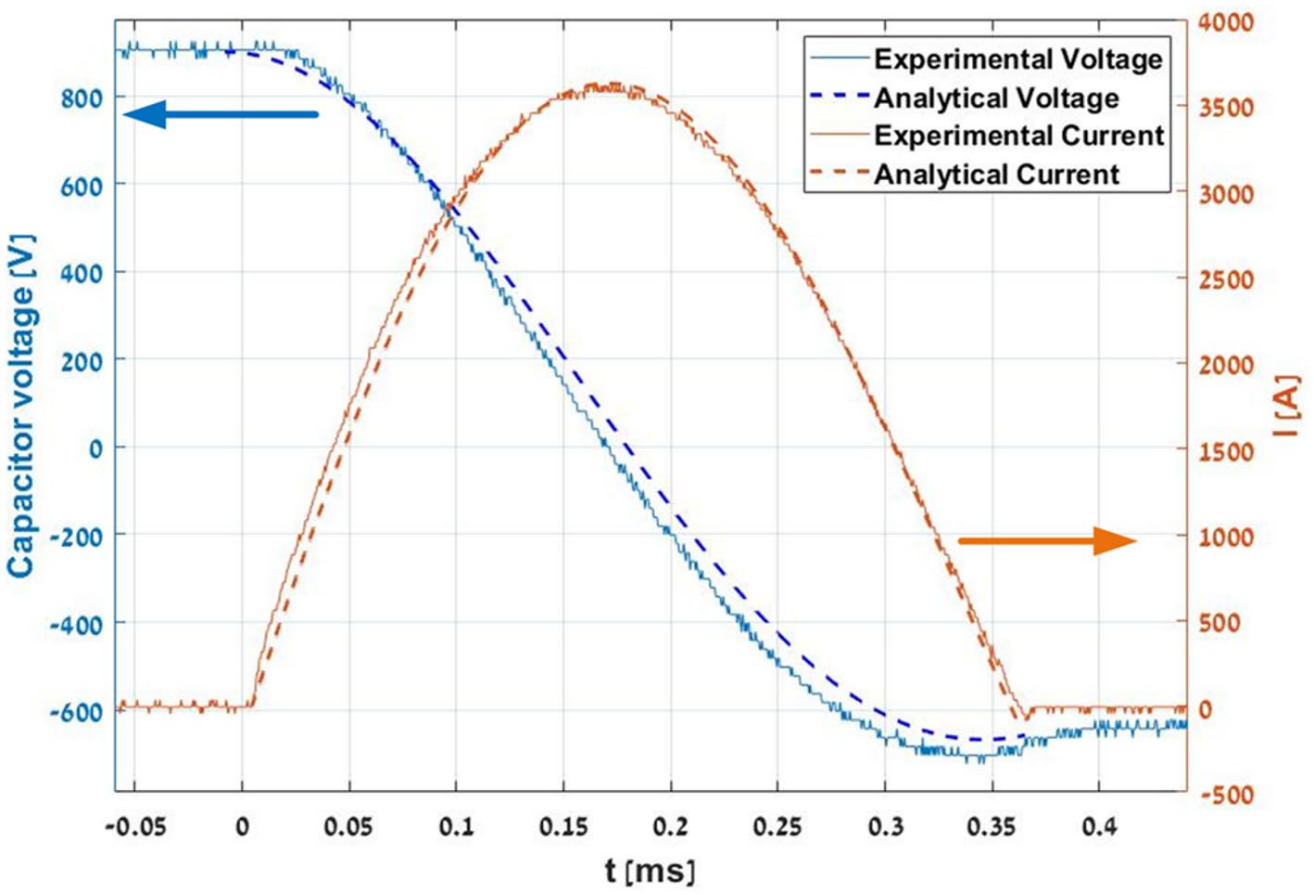
Analytical and experimental results of the capacitor voltage and coil current for the energy recycling ignition circuit with reduced current fall time.

The coil current and capacitor voltage were measured experimentally according to the operating voltage and coil properties that are listed in Table 1. The experimental results are compared with an analytical simulation result and presented in Fig. 4. It can be seen that a maximal current of 3600 A is obtained in a pulse duration of 350 µs. The capacitor is re-charged to a reverse voltage of 650 V, 72% of the initial voltage (900 V). As such, the simulation and measurement are similar.

**Table 1:** The coil and projectile properties.

Properties	
Projectile length [mm]	11
Projectile diameter [mm]	6
Projectile mass [g]	2.5
Coil length [mm]	15.5
Coil inner radius [mm]	3.6
Number of turns	6 × 8 = 48
Wire diameter [mm]	1.5
Coil inductance [µH]	22
Coil resistance [mΩ]	30
Capacitor [µF]	590
Capacitor voltage [V]	900

## Simulation and experiment of the first stage

**Figure 5:**
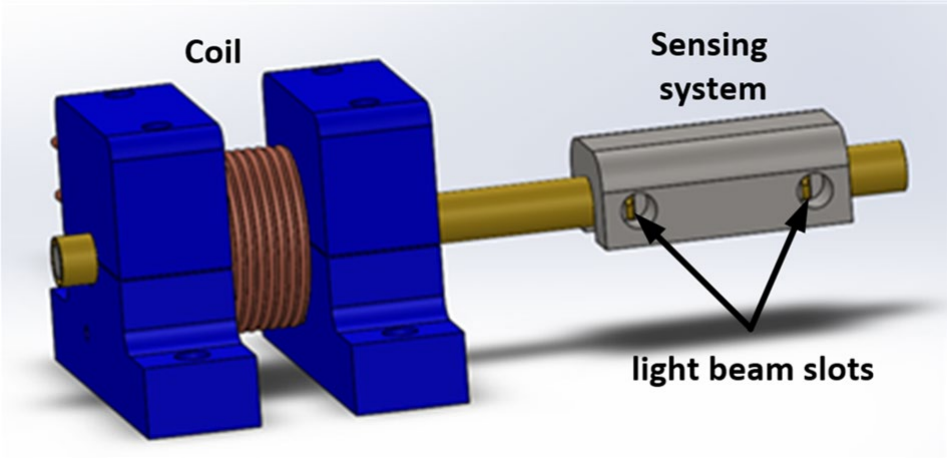
The coil and sensing system schematic illustration.

An elementary coilgun stage was designed with Comsol Multiphysics simulation and tested experimentally. The coil and projectile properties are presented in Table 1, while a schematic illustration of the stage is shown in Fig. 5.

The projectile was made of a 95% cobalt alloy (2 V-Permendur) known for its high saturation magnetic field, B_s_ ~ 2.4 T, and a relatively high permeability. As a consequence, the magnetic force propelling the projectile is improved resulting in velocity and efficiency enhancement^34^.

**Figure 6:**
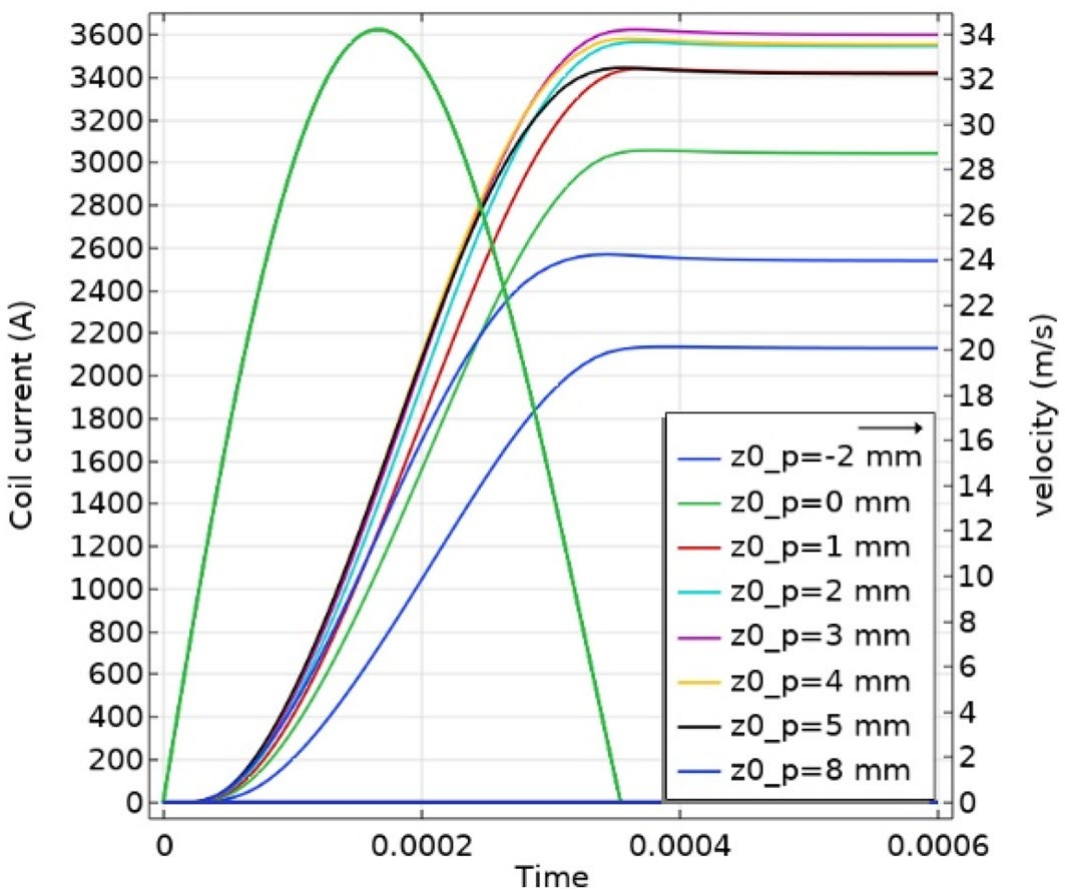
First stage simulation results, a parameter sweep of the optimal initial position (z0_p).

The analytic current pulse presented in Fig. 4 was used for the magnetic simulation in Comsol. The optimal initial position was found by sweeping different positions of the projectile front edge relative to the coil entrance. The simulation results are shown in Fig. 6. It can be seen that the optimal initial position (z0_p) is when the projectile front edge is 3 mm into the coil.

**Figure 7:**
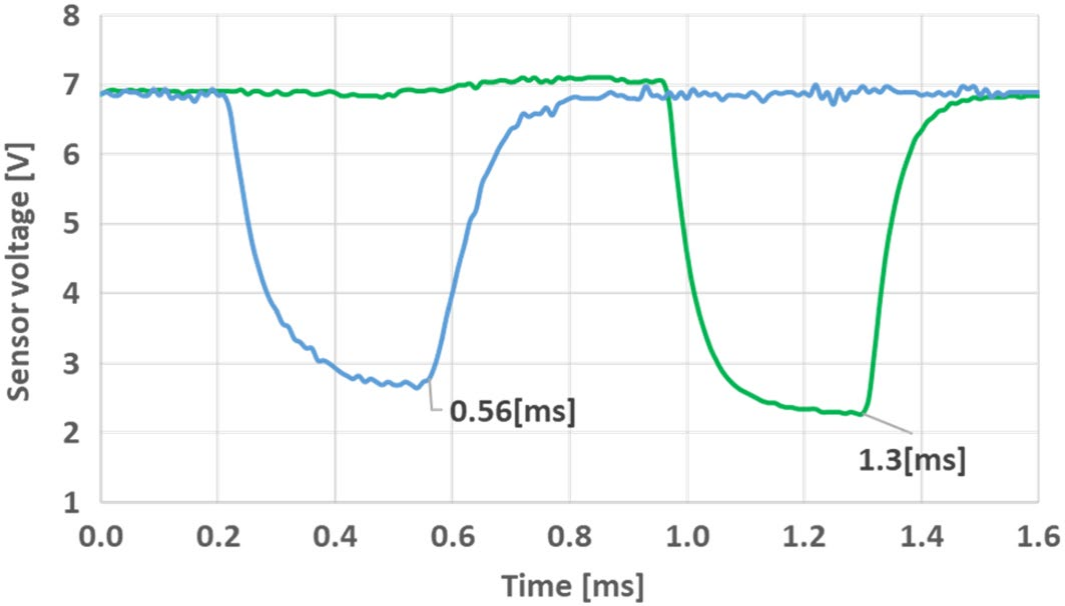
Sensing voltage detection of the first stage velocity, represents velocity of 33.8 m/s.

Based on the results above, the first stage was experimentally tested in the same initial position points. For each initial position, the projectile velocity was measured by a sensing system which includes two pairs of Light Emitting Diodes (LED) and Phototransistors that are located at fixed distances of 25 mm from each other. When the projectile passes the light beam of each LED, a phototransistors voltage drop is measured in the oscilloscope (Fig. 7). The projectile velocity is calculated from the measured time interval between the voltage drops. Figure 7 shows an example of a velocity measurement for the first stage where a velocity of 33.8 m/s is obtained.

**Figure 8:**
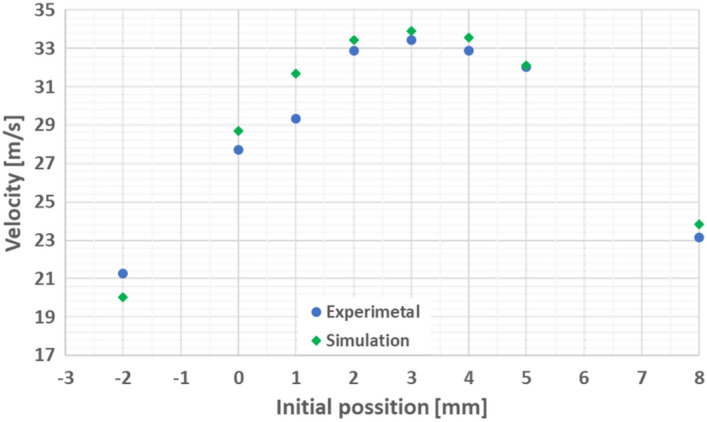
Projectile velocity as function of the initial position for the first stage. Simulation and experiment comparison.

The comparison of the experimental and simulation velocity results for the first stage are presented in Fig. 8. The results show a good accuracy of the simulation in the design and optimization of the system. According to these results, the initial position of 3 mm into the coil was selected as the optimal initial position.

## Simulation and experiment of a multi-stage system

**Figure 9:**
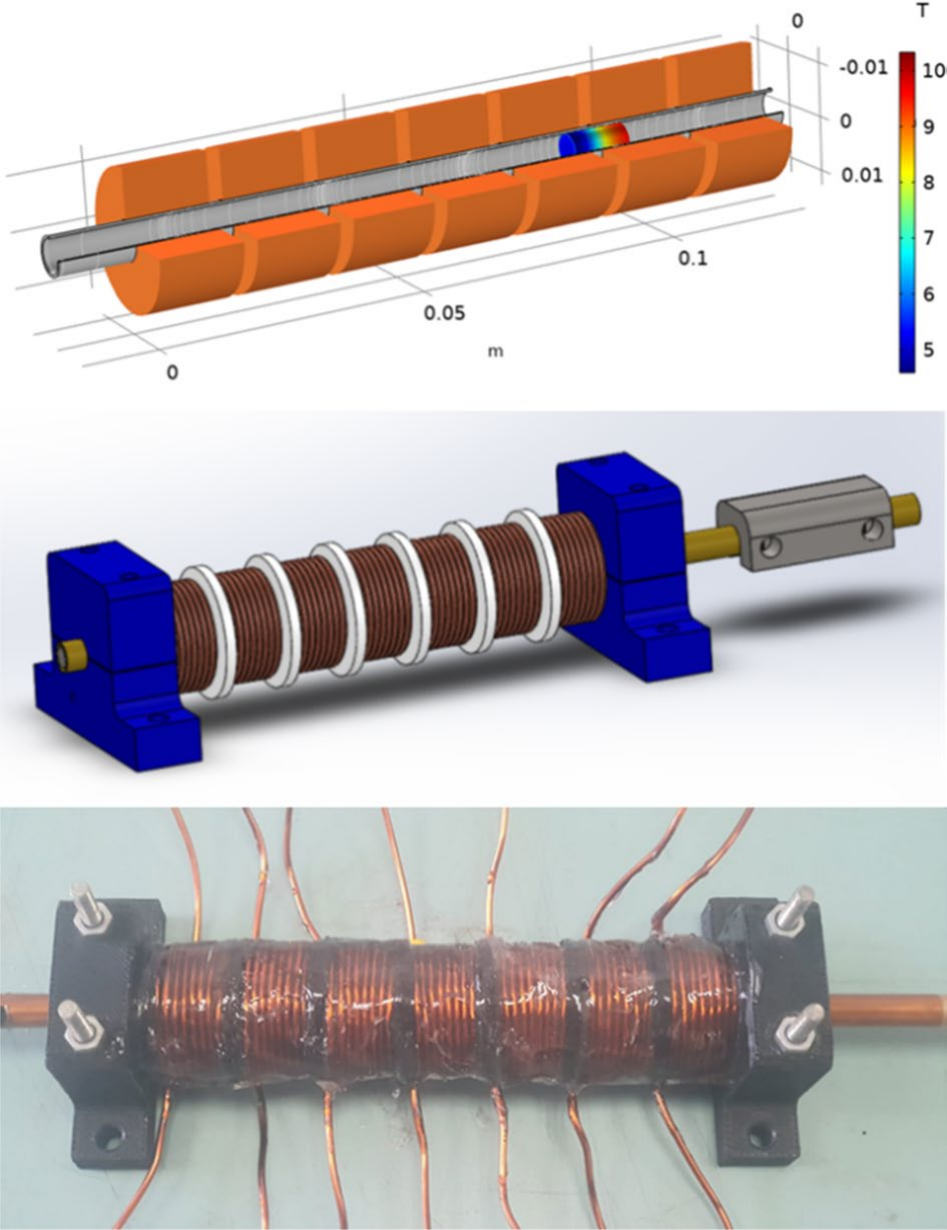
Structure of the 7 stage coilgun, the electromagnetic simulation, the mechanical model, and a picture of the constructed system.

The objective of this section is to simulate the multi-stage system and test it experimentally. The first stage coil was replicated for a total of seven stages distant 2.5 mm from each other. The multi-stage system was tested first in simulation. According to the result a mechanical model was designed and constructed as seen in Fig. 9.

**Figure 10:**
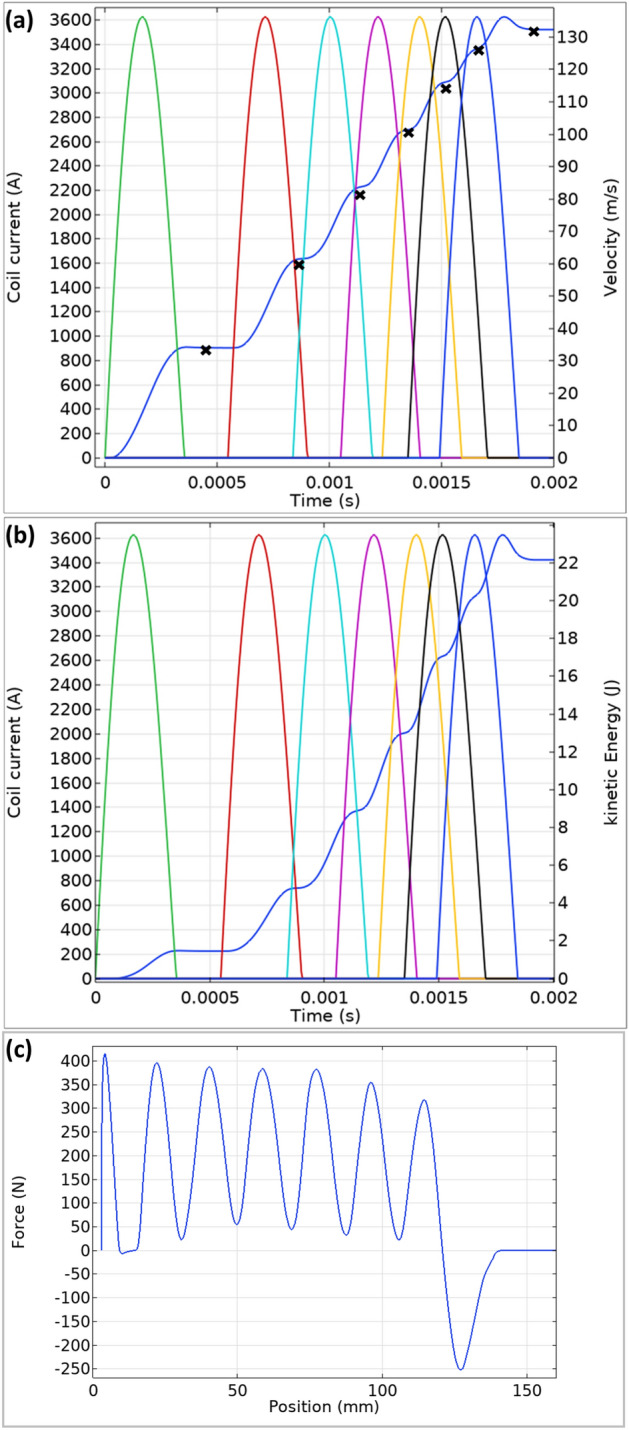
Simulation results of seven stage launcher, velocity (**a**) and energy (**b**) as a function of time, and force as a function of the position (**c**). The '**x**' marks represent the experimental measurements results.

Cascading multi-stages requires careful timing of the current pulses. Therefore, a parameter sweep of the delay between the current pulses was carried out. The delay was determined by sweeping the current turn on time and choosing the optimal delay. Figure 10a shows a simulated 132.3 m/s velocity for a seven stage launcher, while Fig. 10b shows a 22.1 J kinetic energy developed along the stages, which is directly calculated from the simulated velocity as 0.5 mV^2^. It can be seen that every stage adds velocity and energy to the projectile. The force accelerating the projectile is presented in Fig. 10c as a function of the projectile position.

**Figure 11:**
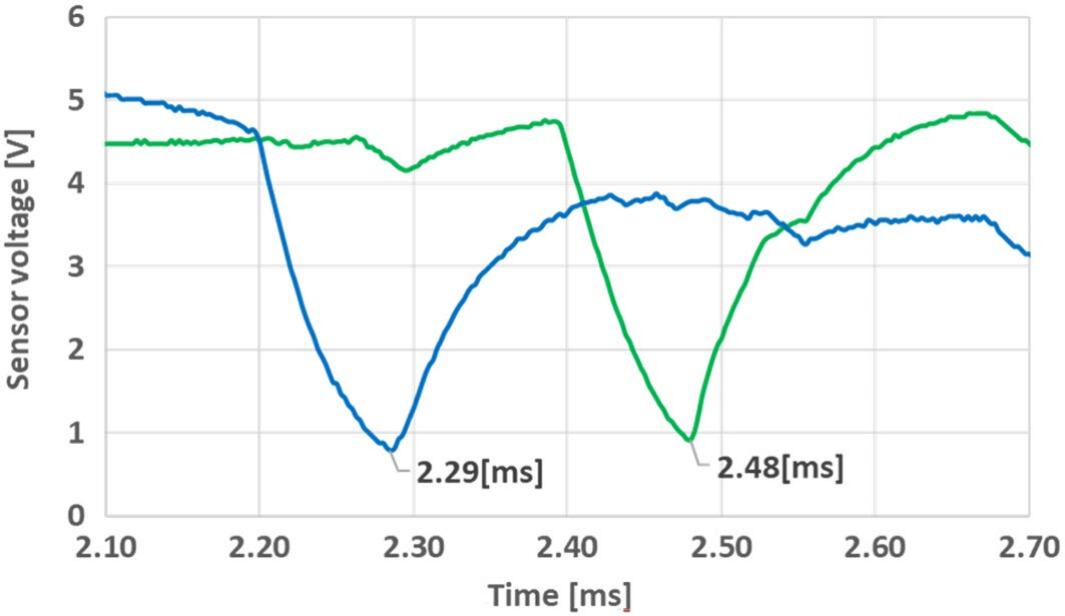
The sensing voltage detection for seven stage launching velocity, represents velocity of 131.5 m/s.

To experimentally verify the simulation, six similar additional stages were included in the experiment. The electrical circuit was also replicated and the 7 triggers of the stages were connected through drivers to a timing computer, where various timing for the triggers can be programmed. The timing intervals were taken from the simulation as detailed in Table 2. Once the projectile was located in the initial position, a series of triggers were given in the pre-ordered sequence. The velocity was measured after the activation of every stage. The results are presented as '**x**' marks in Fig. 10a. The final launching velocity of the entire 7 stages was measured as can be seen in Fig. 11. The projectile passing time between the optical sensors is 0.19 ms and the related velocity calculated as 25 mm/0.19 ms is 131.5 m/s, an error of less than 1% relative to the simulation results.

**Table 2:** Timing of the stages trigger.

Stage number	#1	#2	#3	#4	#5	#6	#7
Trigger time [ms]	0	0.55	0.84	1.05	1.24	1.36	1.50

## Conclusion

This work presents a design of a multi-stage electromagnetic launching system concept, where stages can be added to obtain higher and higher launching speed. An electrical circuit with energy recycling has contributed to achieve the highest velocity measured by reluctance launcher. The stages are simulated correctly and the experiment validated the simulated system. In this concept the acceleration is divided to many smaller stages that are easier to assembly in a modular manner. The result in this experiment is the highest reported launching velocity with a reluctance coilgun, 131 m/s.

The presented concept can be adopted to reach higher velocities by extending the number of stages. It should be noted that added energy is not uniform among the stages. As seen in Fig. 10b, the highest energy gain is in stages 3 and 4. The suggested explanation is the relation between the velocity and the coil geometry. Careful analysis of these stages can lead to further improvements. There are many parameters in a multi-stage coilgun system. Based on empirical experiments and simulations practical design method is to choose a coil length that is between the projectile length to twice the projectile length. The current pulse duration should be short enough to avoid pull back of the projectile, so the electrical LC circuit should support this requirement. The wire diameter must support the current pulse to avoid fusing. In the experiment co-depended electrical parameters are the wire diameter, inductance, resistance, capacitance, and voltage. Once an initial set of parameters is fixed, each parameter is swept by simulation to find an optimal value.

The ultimate launching velocity for the multi-stage coilgun has yet to be reached and currently the limitations of this concept are not clear. A much higher launching velocity may be practical, and, since it is an electrical device, harnessing this concept for space and moon launching is desirable. The reluctance acceleration has the benefits of projectile simplicity and longer lifetime of the launcher. Together with the high-speed launching potential, the multi-stage coilgun is a promising candidate for space and moon missions.

## Data Availability

All data generated or analysed during this study are included in this published article.
